# Hospital Malnutrition, Nutritional Risk Factors, and Elements of Nutritional Care in Europe: Comparison of Polish Results with All European Countries Participating in the nDay Survey

**DOI:** 10.3390/nu13010263

**Published:** 2021-01-18

**Authors:** Joanna Ostrowska, Isabella Sulz, Silvia Tarantino, Michael Hiesmayr, Dorota Szostak-Węgierek

**Affiliations:** 1 Department of Clinical Dietetics, Faculty of Health Sciences, Medical University of Warsaw, E Ciołka Str. 27, 01-445 Warsaw, Poland; dorota.szostak-wegierek@wum.edu.pl; 2 Institute for Medical Statistics, Center for Medical Statistics, Informatics and Intelligent Systems, Medical University Vienna, 1090 Vienna, Austria; isabella.sulz@meduniwien.ac.at (I.S.); silvia.tarantino@meduniwien.ac.at (S.T.); michael.hiesmayr@meduniwien.ac.at (M.H.)

**Keywords:** nutrition risk factors, malnutrition, nutritional care

## Abstract

NutritionDay (nDay) is a project established by the Medical University of Vienna and the European Society for Clinical Nutrition and Metabolism (ESPEN) to audit the nutritional status of hospitalized patients and nursing home residents. This study aimed to evaluate nDay data describing the prevalence of hospital malnutrition, nutritional risk factors, and elements of the nutritional care process implemented in hospital wards in 25 European countries and to compare the data derived from Poland with the data collected in all the European countries participating in the study. In total, 10,863 patients (European reference group: 10,863 participants including Poland: 498 participants) were involved in the study. The prevalence of malnutrition was identified on the basis of the ESPEN diagnostic criteria established in 2015, while the prevalence of nutritional risk factors was assessed by analyzing the following parameters: body mass index (BMI), score of Malnutrition Screening Tool (MST), recent weight loss, insufficient food intake, decreased appetite, increased number of drugs intake, reduced mobility, and poor self-reported health status. Malnutrition prevalence was 12.9% in patients from the European reference group and 9.4% in patients from Polish hospital wards (*p* < 0.05). However, the prevalence of some nutritional risk factors, i.e., recent weight loss, history of decreased food intake, and low actual food intake, were approximately four times more prevalent than diagnosed malnutrition (referring to approximately 40–50% of all participants). In comparison to the European reference group, the significant differences observed in Polish hospital wards concerned mainly dietitian’s involvement in the process of treating malnutrition (16% vs. 57.2%; *p* < 0.001); supply of special diets (8% vs. 16.1%; *p* < 0.0001); provision of oral nutritional support (ONS) (3.8% vs. 12.2%; *p* < 0.0001); prescription of enteral/parenteral nutrition therapy to hospitalized patients (8.2% vs. 11.7%; *p* < 0.001); as well as recording patient weight performed at hospital admission (100% vs. 72.9%; *p* < 0.0001), weekly (20% vs. 41.4%; *p* < 0.05), and occasionally (0% vs. 9.2%). These results indicate that the prevalence of malnutrition and malnutrition risk factors in hospitalized patients in Poland was slightly lower than in the European reference group. However, some elements of the nutritional care process in Polish hospitals were found insufficient and demand more attention.

## 1. Introduction

Malnutrition has been identified as a cause of increased complications which result in longer hospitalization, longer recovery periods, and higher mortality [1,2,3,4]. In hospital settings, malnutrition is frequently observed in elderly patients and patients with chronic and acute diseases. The origin of malnutrition is often multifactorial. It is directly caused by poor nutrient intake, reduced nutrient bioavailability, and high requirements. Consequently, patients often do not meet their daily need for energy, protein, and other nutrients [5]. In addition, other significant determinants of malnutrition reported in the studies are poor appetite, dysphagia, inflammation, malabsorption, age, polypharmacy (6–9 drugs), excessive polypharmacy (10+ drugs), reduced mobility, and poor self-reported health status [5,6,7].

Malnutrition is considered a separate disease entity and therefore has its own code in the International Classification of Diseases (ICD) [8]. As with all diseases, malnutrition should be treated according to current guidelines [9,10,11]. Recent results from a large randomized study by Schuetz P. et al. [12] demonstrated that implementation of individualized nutritional support already in the case of patients identified as being at risk of malnutrition improved important clinical outcomes including decreased short- and long-term mortality. These findings fully correspond to the concept of systematically performed screening of patients on hospital admission and draw attention to the fact that early diagnosis of malnutrition or risk of malnutrition should be considered a priority for medical staff.

According to European Society for Clinical Nutrition and Metabolism (ESPEN) criteria for diagnosing malnutrition established in 2015, patients should be first assessed on the basis of a formal, validated screening tool for the risk of malnutrition. In the group of patients classified as subjects at risk, it was recognized that a BMI value below 18.5 kg/m² is sufficient to diagnose malnutrition. However, if BMI exceeds this value, one of two other methods of diagnosing malnutrition must be used. They should include a combination of reported unintended weight loss and a simultaneous low value of at least one of the following indices: BMI or fat free mass index (FFMI) [13].

NutritionDay (nDay) is a worldwide project aiming to improve the awareness of malnutrition in medical facilities. It is a one-day, annual study—first conducted in 2006—using standardized questionnaires and performed worldwide in hospitals (all types of hospital wards), nursing homes, and intensive care units. In the course of all these years, the survey collected a lot of comprehensive data about the nutritional status of patients and elements of the nutritional care process in medical facilities in many countries all over the world [14].

The aim of this study is to present and evaluate collected nDay data describing the prevalence of hospital malnutrition, nutritional risk factors, and elements of the nutritional care process implemented in hospital wards in 25 European countries and to compare the data derived from Poland with the data collected in all European countries participating in the study.

## 2. Materials and Methods

### 2.1. Study Participants

The overall study included data from 649 European hospital units, which refers to 10,863 patients. All of 10,863 patients were from European countries participating in the nDay survey—referred as the “European reference group”—including 498 patients who were hospitalized in Polish hospital units. The “European reference group” consists of selected 25 European countries (according to World Health Organization categorization): Belgium (21%), Czech Republic (20%), Austria (19%), Portugal (8%), Germany (6%), Poland (5%), Spain (3%), Greece (2%), Croatia (2%), Israel (2%), Italy (2%), Lithuania (2%), and Norway (2%), as well as Bulgaria, Switzerland, Cyprus, Denmark, Estonia, Finland, France, Georgia, Great Britain, The Netherlands, Sweden, and Slovenia, with each less than 100 patients (<1%).

All subjects gave their informed consent for inclusion before they participated in the study. The worldwide coordinating center at the Medical University Vienna gains yearly ethical approval for multicenter data collection. The study is registered at the clinicaltrials.gov with the identifier NCT02820246.

### 2.2. Study Design and Processes

nDay is a one-day, annual, cross-sectional, multicenter audit which is performed worldwide in hospitals, nursing homes, and intensive care units on one given day per year.

Each unit participates in the audit on a voluntary and anonymous basis by online registration. Data collection and its entry to the nDay database were performed at the ward level by medical staff members using online available questionnaires (www.nutritionDay.org) [15]. All patients hospitalized from 7 a.m. to 7 p.m. on the day of conduction for nDay were included in the audit (including admissions and discharges within the period). The exclusion criteria covers patients under 6 years of age, patients unable to answer questions, and patients admitted and discharged from a hospital unit on the same day.

This manuscript is based on data derived from hospitals from 25 European countries which participated in the nDay study in 2015.

### 2.3. Nutritional Risk Factors

The prevalence of nutritional risk factors was assessed by analyzing the parameters described in Table 1.

### 2.4. Diagnose of Malnutrition

The prevalence of malnutrition was assessed by mapping questions from nDay questionnaires to the ESPEN diagnostic criteria for malnutrition established in 2015 [13]. The first step was to screen patients for risk of malnutrition—MST [16] was used. Patients who scored 2 points or more were classified as subjects at risk of malnutrition. In the group of patients at risk of malnutrition, the second step of assessment was conducted. A BMI below 18.5 kg/m² was sufficient to diagnose malnutrition. If, however, the BMI exceeded this value, the other method of diagnosing malnutrition was used. It required a combination of reported unintended weight loss and a simultaneous low value of BMI (Algorithm 1).
**Algorithm 1.** Criteria used to diagnose malnutrition based on European Society for Clinical Nutrition and Metabolism (ESPEN) criteria established in 2015 [13].Step 1 Diagnosis of risk of malnutritionMalnutrition Screening Tool (MST) [16] ≥ 2 pointsQuestions and score:*Have you recently lost weight without trying?*no 0/unsure 2*If yes, how much weight have you lost?*2–13 lb 1/14–23 lb 2/24–33 lb 3/34 lb or more 4/unsure 2*Have you been eating poorly because of a decreased appetite?*no 0/ yes 1Step 2 Diagnosis of malnutritionAlternative 1BMI < 18.5 kg/m²orAlternative 2Unintended body weight loss (>5% within the last 3 months or >10% within an unspecified time frame)andBMI < 20 kg/m² for subjects < 70 yearsBMI < 22 kg/m² for subjects > 70 years

### 2.5. Statistical Analysis

Data was analyzed using R 3.6.3. Results were shown in frequencies with percentages, median with interquartile range (IQR) or mean and standard deviation (SD) when appropriate. To check whether differences are significant, *t*-Test, Wilcoxon-U-Test, Chi-squared Tests, or Fisher Exact Test were used where appropriate (*t*-Test for mean + SD, Wilcoxon-U-Test for median + IQR, Chi-squared Tests for proportions, and Fisher Exact Test for proportions when *n* < 5). In cases where computational problems calculating Fisher’s Exact test appeared, the *p*-value was computed by using Monte Carlo simulation. *p* < 0.05 was assumed as the level of statistical significance. All data analysis was done at the Department for Medical Statistics, Medical University Vienna.

## 3. Results

### 3.1. Subjects’ Characteristics and Demographics

From the European reference group, 10,863 patients hospitalized in 649 wards of 19 different specialties were included in the study. Patients were approximately equally divided by gender, with a slight majority of female participants. The median age of the patients was 70 years (IQR 56–80). The average BMI was about 26 kg/m², while the typical body weight (defined as body weight 5 years prior to conducting the nDay survey) was on average 5 kg higher, which shows almost 7% loss in typical body weight of examined patients (Table 2). The most common comorbidities were related to affected gastrointestinal tract (22.8%) or heart/circulation system (22.5%). Patients’ length of hospital stay prior to conducting the nDay survey was a median of 6 days (IQR 3–12). The largest group of patients in the nDay database was hospitalized in two medical specialties: general internal medicine (14.3%) and general surgery (13.9%) (Table 3).

In Poland, the nDay audit referred to a total of 498 patients who were treated in 25 units. Data from Polish facilities show that the median age of patients was on average 6 years lower (64 years (IQR 52–76)) than in the European reference group and that the average patients’ length of hospital stay prior to conducting the study was shorter—a median of 4 days (IQR 2–10). Additionally, in Polish hospital wards, there were significantly lower numbers (*p* < 0.0001) of cancer patients and patients with infections and with disorders of the locomotor system. At the same time, more patients (*p* < 0.0001) with endocrine diseases and blood disorders were identified in Polish wards. In the case of other characteristics, no significant difference was observed between the national and the European reference group (Table 2).

### 3.2. Subjects’ Nutritional Status

#### 3.2.1. Prevalence of Nutritional Risk Factors

Malnutrition risk (MST score ≥ 2 points) was identified in 30% of patients from the European reference group, whereas analyzed the data revealed that hospital patients were mostly affected by declared unintentional body weight loss and insufficient food intake (these nutritional risk factors referred to about 40–50% of all participants).

**Figure 1:**
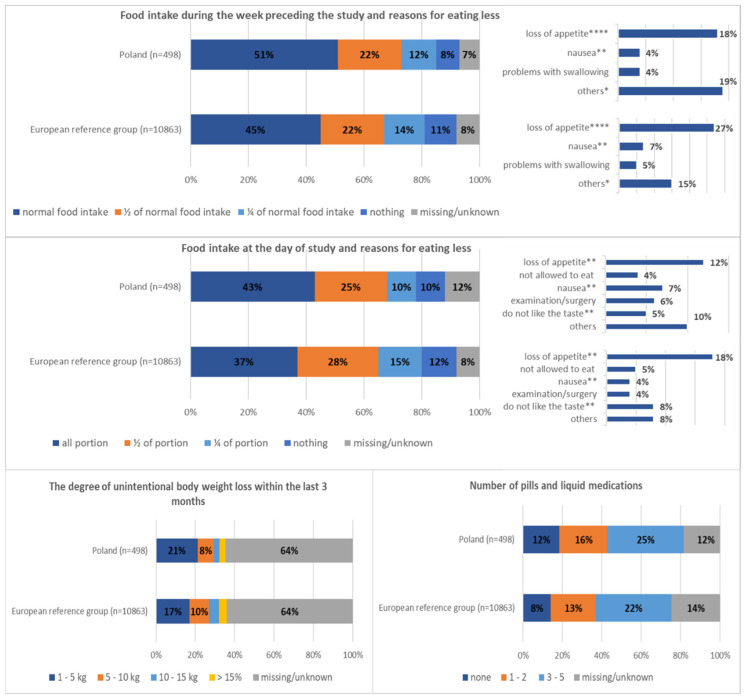
The most prevalent risk factors in detail. * *p* < 0.05, ** *p* < 0.01, and **** *p* < 0.0001.

This similar pattern was noted also for Polish participants—the prevalence of recent weight loss, history of decreased eating, and low actual eating were almost twice more prevalent than malnutrition risk diagnoses based on MST and over six times more prevalent than low BMI (Table 4). The largest group of patients did not answer what was the degree (in kilograms) of weight lost (64% missing data) or declared about 5 kg of unintentional body weight loss within the last 3 months and half of actual food intake (on the day of study) and during the week preceding the study. It is also worth mentioning that about 10% of Polish and European reference group participants declared that they ate nothing on the day of the study and during the week prior the study (Figure 1).

#### 3.2.2. Prevalence of Malnutrition

In the European reference group, 1406 (12.9% of all participants) out of the 10,863 patients were classified as malnourished according to ESPEN diagnostic criteria. However, it should be noted that certain data relative to 15.7% of patients were missing. Consequently, the classification of the abovementioned data was not feasible. The proportion of malnourished patients in almost 80% was identified on the basis of the partial ESPEN criteria referred as “alternative 2”.

**Figure 2:**
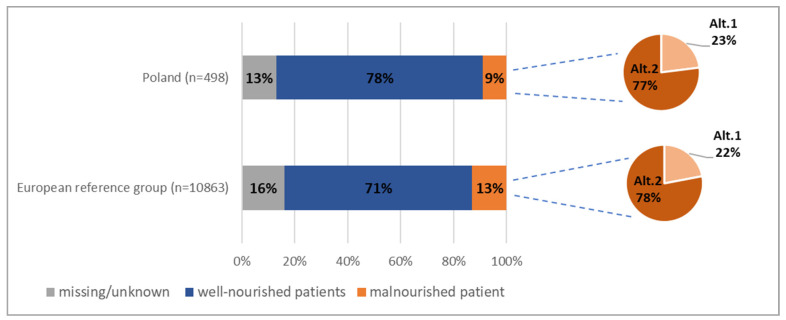
Prevalence of malnutrition in percentage. Abbreviations: Alt. 1, Alternative 1; Alt. 2, Alternative 2.

In Polish hospital wards, the percentage of malnourished patients was significantly lower than in the case of the European reference group (9.4% vs. 12.9%; *p* < 0.05) (Table 5, Figure 2).

### 3.3. Elements of Nutritional Care Process

#### 3.3.1. Food Provision/Nutrition Support Offered to Patients

Oral diet, either normal hospital food (*n* = 7386, 68%) or special diet (*n* = 1745, 16.1%), was mostly supplied to all patients. Oral nutritional support (ONS) was provided to 12.2% (*n* = 1325), whereas enteral/parenteral nutrition therapy was prescribed to 11.7% (*n* = 1276) of patients from the European reference group.

The percentage of Polish patients receiving ONS was significantly lower than in the European reference group (3.8% vs. 12.2%; *p* < 0.0001). The same was noted in the case of prescription of special diet and enteral/parenteral nutrition (Table 6).

#### 3.3.2. Nutrition Staffing in the Hospital/Ward

A vast majority of the units from the European reference group declared that a nutrition support team and nutritional care persons exist in the hospital or in the hospital ward.

The abovementioned nutrition staff was noted in high percentage also in Polish facilities, including 100% (*n* = 25) of units declaring the presence of a support team in their hospitals. In the case of other healthcare professionals indicated in Table 6, no significant difference was observed between Poland and the European reference group except for the higher median value of physicians working morning shift in the Polish hospital wards (4 [2–6] vs. 2 [1–4]; *p* < 0.01).

#### 3.3.3. Nutrition Guidelines/Screening Structures

In the European reference group, recording patients’ weight and nutritional screening was most often performed when patients were admitted to the hospital (72.9%) while weekly and occasional weighing of patients were carried out much less frequently.

According to the caregivers, all Polish patients were weighed and examined for malnutrition during admission to the hospital. On the other hand, no occasional weighing of patients was declared in any Polish unit and the percentage of wards declaring weekly recording patients’ weight was twice lower when compared to the European reference group (20% vs. 41.4%; *p* < 0.05) (Table 6).

Most frequently, all European patients were screened using one of the formal, validated screening tool: Nutritional Risk Screening (NRS 2002) (Poland: 92%; European reference group: 43.9%). Additionally, in the case of the European reference group, beside NRS 2002, nutritional screening was most often conducted using the experience of healthcare professionals (36.2%) and locally developed tools (27.1%) (Table 6).

#### 3.3.4. Structures in the Wards Managing Malnourished/at Risk of Malnutrition Patients

Most of the units included in the European reference group consulted a dietician (57.2%) and developed an individual nutrition care plan (47.8%) in order to cope with a problem of malnutrition or risk of malnutrition.

**Figure 3:**
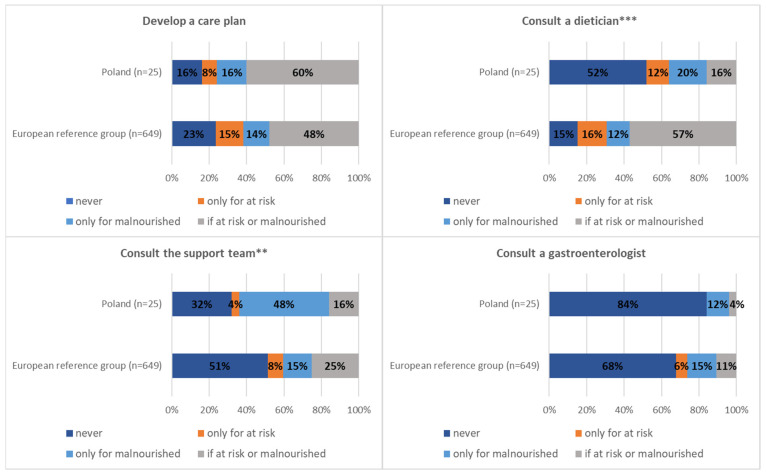
Structures in the wards managing malnourished/at risk of malnutrition patients in detail. ** *p* < 0.01 and *** *p* < 0.001.

Most often, Polish caregivers declared that developing an individual nutrition care plan is an intervention undertaken in cases of diagnosis of malnutrition or risk of malnutrition (60%). However, only 16% of hospital units consulted a dietician when the patient was malnourished or at nutritional risk—this percentage is nearly four times lower than data observed in the case of the European reference group (16% vs. 57.2%, *p* < 0.001). Moreover, a nutrition expert (dietician/nutritional support team/gastroenterologist) has been never consulted in more than 16% of cases of malnourished patients or at risk of malnutrition (Table 6 and Figure 3).

## 4. Discussion

This study is one of the first to analyze the prevalence of hospital malnutrition according to ESPEN diagnostic criteria established in 2015 on such a large group of international patients. In the European reference group (*n* = 10,863), 12.9% of all participants were classified as malnourished, while in Polish hospital wards (*n* = 498), the percentage of malnourished patients was 9.4%. It should be emphasized that approximately 15% of data in each studied group was missing. The lack of this data partially precludes the classification of patients as malnourished. Therefore, it cannot be excluded that the percentage of malnourished patients was slightly higher than that presented in the study results.

Nevertheless, similar results were obtained in a Greek study by Poulia KA. et al. [17], who reported malnutrition in approximately 11% of 362 hospitalized patients with the use of the same ESPEN diagnostic criteria. A Portuguese study by Guerra RS. et al. (*n* = 782) [18] showed that approximately 12% were malnourished, while Orlandoni P. et al. [19] reported a higher result obtained also on the basis of ESPEN criteria (*n* = 284), where malnutrition was reported in approximately 25% of participants. It is worth noting that the last study was conducted in a group of elderly patients, which may explain a higher percentage of malnourished patients.

The proportion of malnourished patients in almost 80% was identified with the use of partial criteria referred to as “alternative 2”, including a combination of reported unintended weight loss and a simultaneous low value of BMI depending on patient’s age (less than 22 kg/m² for subjects over the age of 70 years and less than 20 kg/m² for the rest of the patients). This is understandable considering the fact that the process of aging is associated with a reduction of muscle mass (sarcopenia) and a relative increase of fat tissue. The coexistence of sarcopenia with obesity leads to a state called “sarcopenic obesity” and consequently results in even higher values of BMI [20,21]. Nevertheless, the reduction of lean body mass (sarcopenia) of patients has a higher predictive value for the duration of hospitalization, frequency of rehospitalizations, and the incidence of complications [22]. Moreover, in the case of patients with chronic diseases, mortality is lower with increased lean body mass and even obesity [23,24]. This observation is called the “obesity paradox”.

The prevalence of malnutrition risk factors was significantly higher than the prevalence of malnutrition. Malnutrition risk (MST score ≥ 2 points) was identified for 30% of patients from the European reference group and for 25% of Polish participants. Moreover, prevalence of recent weight loss (Poland: 39%; European reference group: 41%), history of decreased eating (Poland: 41%; European reference group: 46%), and low actual eating (Poland: 46%; European reference group: 54%) were approximately four times more prevalent than diagnosed malnutrition, twice more prevalent than malnutrition risk identified based on MST, and over six times more prevalent than BMI <18.5 kg/m² (Table 4). Furthermore, it should be noted that, in the group of patients who declared body weight loss (Table 4), the majority of data (64%) on the degree (in kilograms) of this loss was missing (Figure 1). Most probably, it might be a result of a lack of respondents’ knowledge, while knowing the degree of patients’ weight loss is one of important factors in a process of diagnosing malnutrition [13]. Therefore, to prevent malnutrition in inpatients, there seems to be a need to establish a system for recognizing patients’ weight loss not only by weighing patients regularly during hospitalization but also by recommending regular weight record at home. Further, it should be highlighted that this similar pattern of high prevalence of malnutrition risk factors was noted for all studied patients and indicated that increased attention should be given to all abovementioned parameters. Recent results from a large randomized trial published in *The Lancet* journal demonstrated that nutrition care processes implemented for patients with nutrition risk factors is associated with decreased mortality [13]. Moreover, this kind of intervention may be less complex and costly and cam potentially lead to reduced hospital length of stay and better outcomes of hospitalized patient [25].

Summarizing the assessment of nutritional status of the studied patients, it should be noted that the prevalence of malnutrition and malnutrition risk factors of hospitalized patients in Poland are slightly lower than in the case of the European reference group. This may be explained by the fact that the median age of Polish patients was lower and the average patient length of hospital stay was shorter than in the European reference group. These two determinants (older age and longer hospital stay in European reference group) are associated with increases prevalence of malnutrition, a conclusion formed from many studies [5,7,26]. Moreover, in Polish hospital wards, there were significantly lower numbers of cancer patients participating in the study, which could be related to the large number of surgical patients and the fact that adequate nutritional status of a patient is a condition which should be fulfilled before carrying out surgery [27]. Perioperative nutrition is a fundamental and integral part of perioperative care and has been included in many Enhanced Recovery After Surgery (ERAS) programs which aim to decrease the postoperative complications [28]. ERAS Society guidelines recommend ONS provision before many types of major surgery in patients at risk of malnutrition [29,30,31].

The large commitment of medical staff in the process of nutrition care is a requirement of obtaining positive outcomes in a hospitalized patient. The nutrition support team includes physicians, nurses, and dieticians (responsible for nutritional assessment, diet ordering, and documentation) as well as physiotherapists who monitor the results of the therapy, speech therapists coping with swallowing disorders, and pharmacists involved in clinical nutrition supply [25]. However, based on the conducted study, in Polish hospital wards, a nutrition expert (dietician/nutrition support team/gastroenterologist) has been never consulted in more than 16% of cases of malnourished or at risk of malnourishment patients. This is particularly evident in the involvement of a dietician being four times lower than in the case of the European reference group. This low commitment of members of the nutrition support team in the process of nutrition care in Polish medical units may explain the lower supply of special diets (8% vs. 16.1%), provision of ONS (3.8% vs. 12.2%), and prescription of enteral/parenteral nutrition therapy to hospitalized patients (8.2% vs. 11.7%). Moreover, the lower supply of ONS can be caused by its reduced availability (due to no refund) in Polish medical facilities compared to European reference group [32].

The process of diagnosing malnutrition starts with a screening test used to identify patients as malnourished or as patients at increased risk of becoming malnourished. In Poland, according to Ministry of Health regulations dated 15 September 2011, the assessment of nutritional status should be obligatorily performed for each patient during hospital admission [33]. The abovementioned fact is reflected in the results of the study—all Polish patients were examined for malnutrition during admission to the hospital, which was a higher result than in the case of the European reference group (100% vs. 72.9%). This may prove the knowledge of Polish caregivers on the importance of conducting a screening assessment of nutritional status and its legal regulations. On the other hand, no occasional weighing of patients was declared in any Polish unit and the percentage of wards declaring weekly recording of patients’ weights was twice lower when compared to the European reference group. Consequently, it can be assumed that recording patient weight is not performed routinely in Polish wards, and it is necessary to draw attention to this problem and to implement changes in hospital practices.

Some limitations of the present study need to be kept in mind when interpreting the data. First, participating units could not be representative of all hospitals from countries taking part in the study. Participation in the nDay study is voluntary, and recruitment is promoted mainly by nutrition experts. This could result in the participation of medical units with a special interest in nutritional care. Furthermore, such extensive databases including data from 25 countries are burdened with a risk of missing data and non-homogeneous data reporting. Additionally, patients who lacked capacity and those with severe communication problems as well as acute or chronic confusion may not be able to provide consent to participate in the study and consequently were excluded from the studied group. It is possible that those excluded patients were more likely to be nutritionally at risk or malnourished.

## 5. Conclusions

Based on the study results, malnutrition diagnosed in accordance with the ESPEN guidelines established in 2015 applies to a relatively low percentage of hospitalized patients (lower than 15%). However, the prevalence of malnutrition risk factors was significantly higher—increased attention should be given especially to unintended body weight loss, history of decreased food intake, and insufficient actual eating. Moreover, due to an observed lack of patients’ knowledge concerning the degree of recent weight loss, there seems to be a need to establish a system for recognizing weight loss by weighing patients regularly during hospitalization as well as by recommending regular weight recording at home.

The prevalence of malnutrition and malnutrition risk factors of hospitalized patients in Poland are slightly lower than in the case of the other European countries participating in the nDay survey. Nevertheless, elements of the nutritional care process were found insufficient and should be improved. This applies mainly to the involvement of nutrition experts (especially dietician) in the process of treating malnutrition and hence decreased supply of special diets, ONS, and enteral/parenteral nutrition to hospitalized patients as well as a lack of recording patient weight performed weekly and occasionally in Polish hospital wards.

The nDay survey should be continued on both the international and national levels to identify and implement changes in medical practice and to further decrease the prevalence of malnutrition and nutritional risk factors in the hospital setting.

## Figures and Tables

**Table 1:** Criteria used to determine the prevalence of nutritional risk factors.

Nutritional Risk Factor	Malnutrition Risk Criteria
Body mass index (BMI)	<18.5 kg/m²
Unintentional body weight loss within the last 3 months	any body weight loss
Insufficient food intake during the week preceding the study	less than ½ of normal food intake
Insufficient food intake at the day of study	less than ½ of typical portion
Malnutrition Screening Tool (MST) [16]—described below	Score ≥ 2
Decreased appetite	Declared
Number of drugs and liquid medications	>5
Unable to walk without assistance	Declared
Poor self-reported health status	Declared

**Table 2:** Subjects’ characteristics including demographics of the study population.

Characteristic		Poland	European Reference Group	*p*-Value
		*n* = 498	*n* = 10,863	
		Mean (SD)/Median [IQR]	Mean (SD)/Median [IQR]	
Age	Years	64 (52–76)	70 (56–80)	<0.0001
Weight	Kg	73.8 (18.4)	73.3 (18.9)	Ns
Height	Cm	167 (10.5)	167 (12)	Ns
BMI	Kg/m²	26.3 (5.8)	26.1 (5.9)	Ns
Weight 5 years ago (typical weight)	Kg	76.7 (18.4)	78.1 (18.9)	Ns
Duration after hospital admission	Days	4 (2–10)	6 (3–12)	<0.001
		***n* (%)**	***n* (%)**	
Gender	Female	253 (50.8%)	5662 (52.1%)	Ns
	Male	245 (49.2%)	5201 (47.9%)	Ns
Affected organs (multiple answers possible)	Brain, nerves	53 (10.6%)	1678 (15.4%)	<0.01
	Eye, ear	13 (2.6%)	264 (2.4%)	Ns
	Nose, throat	15 (3%)	313 (2.9%)	Ns
	Heart, circulation system	132 (26.5%)	2440 (22.5%)	<0.05
	Lung	70 (14.1%)	1552 (14.3%)	Ns
	Liver	16 (3.2%)	681 (6.3%)	<0.01
	Gastrointestinal tract	103 (20.7%)	2481 (22.8%)	Ns
	Kidney/urinary tract	60 (12%)	1526 (14%)	Ns
	Endocrine system	78 (15.7%)	958 (8.8%)	<0.0001
	Skeleton/bone/muscle	55 (11%)	2234 (20.6%)	<0.0001
	Skin	5 (1%)	406 (3.7%)	<0.01
	Ischaemia	5 (1%)	242 (2.2%)	Ns
	Cancer	87 (17,5%)	2036 (18.7%)	<0.0001
	Infection	3 (0.6%)	654 (6%)	<0.0001
	Pregnancy	0 (0%)	20 (0.2%)	-
	Others	39 (7.8%)	699 (6.4%)	Ns

Ns—no statistical significance.

**Table 3:** Medical specialties included in the study.

	Poland	European Reference Group	*p*-Value *
**Medical speciality**	*n* = 25	*n* = 649	
	***n* (%)**	***n* (%)**	
General internal medicine	3 (12%)	93 (14.3%)	Ns
General surgery	6 (24%)	90 (13.9%)	Ns
Cardiology	0 (0%)	21 (3.2%)	Ns
Gastroenterology/Hepatology	2 (8%)	61 (9.4%)	Ns
Neurology	1 (4%)	31 (4.8%)	Ns
Infectiology	0 (0%)	5 (0.8%)	Ns
Nephrology	1 (4%)	15 (2.3%)	Ns
Oncology	0 (0%)	63 (9.7%)	Ns
Cardiothoracic surgery	0 (0%)	8 (1.2%)	Ns
Psychiatry	0 (0%)	11 (1.7%)	Ns
Ear Nose Throat (ENT)	1 (4%)	21 (3.2%)	Ns
**Characteristics**	***n* (%)**	***n* (%)**	
Geriatrics	1 (4%)	63 (9.7%)	Ns
Long term care	0 (0%)	13 (2%)	Ns
Trauma	0 (0%)	13 (2%)	Ns
Orthopaedic surgery	2 (8%)	37 (5.7%)	Ns
Gynaecology	0 (0%)	9 (1.4%)	Ns
Paediatrics	1 (4%)	16 (2.5%)	Ns
Neurosurgery	0 (0%)	3 (0.5%)	Ns
Others	7 (28%)	76 (11.7%)	Ns

Ns—no statistical significance * *p*-value was simulated with Monte-Carlo Simulation.

**Table 4:** Prevalence of nutritional risk factors.

	Poland	European Reference Group	*p*-Value
Nutritional risk factor	*n* = 498	*n* = 10,863	
	***n* (%)**	***n* (%)**	
MST score ≥ 2 points	122 (24.5%)	3249 (29.9%)	<0.01
BMI < 18.5 kg/m²	31 (6.2%)	677 (6.2%)	Ns
Insufficient food intake during the week preceding the study (less than ½ of normal food intake)	204 (41.0%)	5036 (46.4%)	<0.05
Insufficient food intake at the day of study (less than ½ of typical portion)	227 (45.6%)	5845 (53.8%)	<0.01
Unintentional body weight loss within the last 3 months	196 (39.4%)	4400 (40.5%)	Ns
Decreased appetite	120 (24.1%)	3032 (27.9%)	<0.01
Number of drugs and liquid medications (more than 5)	178 (35.7%)	4752 (43.7%)	<0.01
Unable to walk without assistance	112 (22.5%)	3685 (33.9%)	<0.01
Poor self-reported health status	129 (25.9%)	2202 (20.3%)	Ns

Ns—no statistical significance.

**Table 5:** Prevalence of malnutrition according to ESPEN diagnostic criteria established in 2015.

	Poland	European Reference Group	*p*-Value
Diagnostic criteria	*n* = 498	*n* = 10,863	
	***n* (%)**	***n* (%)**	
Alt. 1 (MST ≥ 2 points + BMI < 18.5 kg/m²)	11 (2.2%)	305 (2.8%)	Ns
Alt. 2 (MST ≥ 2 points + unintended weight loss + BMI < 20 kg/m² or < 22 kg/m²)	36 (8.6%)	1101 (10.1%)	<0.05
Alt. 1 + Alt. 2—Total malnourished patients according to ESPEN diagnostic criteria.	47 (9.4%)	1406 (12.9%)	<0.05
Impossible to verify (missing data)	66 (13.2%)	1704 (15.7%)	-

Abbreviations: Alt. 1, Alternative 1; Alt. 2, Alternative 2.

**Table 6:** Elements of nutrition care process.

**Nutrition Care Indicators**		**Poland**	**European Reference Group**	***p*-Value**
		*n* = 498	*n* = 10,863	
Food provision/nutrition support offered to patients (multiple answers possible) ***n* (%)**				
Oral diet	normal hospital food	363 (72.9%)	7386 (68%)	<0.05
	special diets	40 (8%)	1745 (16.1%)	<0.0001
Provision of ONS		19 (3.8%)	1325 (12.2%)	<0.0001
Prescription of EN/PN/EN + PN		41 (8.2%)	1276 (11.7%)	<0.001
Number of nutrition supports chosen by caregivers	1	402 (80.7%)	8999 (82.8%)	-
	2	47 (9.5%)	1383 (12.7%)	-
	3	0 (0%)	162 (1.5%)	-
	4	0 (0%)	9 (0.1%)	-
	not answered	49 (9.8%)	310 (2.9%)	-
		**Poland**	**European reference group**	
		*n* = 25	*n* = 649	
Nutrition staffing in the hospital/ward (morning shift) ***n* (%)/median + [IQR]**				
Nutrition support team in the hospital		25 (100%)	542 (83.5%)	<0.05
Nutritional care person in the ward		21 (84%)	502 (77.3%)	Ns
Number of physicians in the ward		4 (2–6)	2 (1–4)	<0.01
Number of nurses in the ward		5 (3–7)	4 (3–5)	Ns
Number of nursing aides in the ward		2 (0–2)	2 (1–2)	Ns
Number of dieticians in the ward		0 (0–1)	1 (0–1)	Ns
Number of physiotherapists in the ward		1 (1–1)	1 (0–2)	Ns
Nutrition guidelines/screening structures ***n* (%)**				
Routine screening at admission		25 (100%)	473 (72.9%)	<0.0001
Screening using validated screening tool		24 (96%)	550 (84.7%)	<0.001
Screening using NRS 2002		23 (92%)	285 (43.9%)	<0.0001
Screening using MUST		1 (4%)	41 (6.3%)	Ns
Screening using local screening tool		0	176 (27.1%)	-
Screening using professional experience		2 (8%)	235 (36.2%)	<0.05
Routine weighing at admission		25 (100%)	473 (72.9%)	<0.0001
Routine weighing every week		5 (20%)	269 (41.4%)	<0.05
Weighing occasionally		0	60 (9.2%)	-
Weighing when requested		14 (56%)	340 (52.4%)	Ns
Nutrition guidelines/screening structures ***n* (%)**				
Develop an individual nutrition care plan		15 (60%)	310 (47.8%)	Ns
Dietician consult in case of malnutrition/risk of malnutrition		4 (16%)	371 (57.2%)	<0.001
Support team consult in case of malnutrition/risk of malnutrition		4 (16%)	164 (25.3%)	<0.01
Gastroenterologist consult in case of malnutrition/risk of malnutrition		1 (4%)	70 (10.8%)	Ns

Abbreviations: ONS, Oral nutrition supplements; EN, Enteral nutrition; PN, Parenteral nutrition; NRS 2002, Nutritional Risk Screening; MUST, The Malnutrition Universal Screening Tool.

## Data Availability

All data presented in this study were from a protected online database of the Center for IT Systems & Communications of the Medical University of Vienna (https://cemsiis.meduniwien.ac.at/) in which neither hospitals nor patients can be identified. Upon submission of a research proposal, anonymized data are shared with researcher. Each proposal is submitted to the supervisory board for approval.
